# Obicetrapib and ezetimibe enhance LDL receptor-mediated VLDL clearance and regress atherosclerosis on atorvastatin background

**DOI:** 10.1016/j.jlr.2026.101028

**Published:** 2026-03-25

**Authors:** José A. Inia, Leo H. Zhang, Nanda Keijzer, Nicole Worms, Anita van Nieuwkoop-van Straalen, Marc Ditmarsch, Mathijs de Kleer, J. Wouter Jukema, John J.P. Kastelein, Michael Szarek, Anita M. van den Hoek, Geurt Stokman, Elsbet J. Pieterman, Hans M.G. Princen

**Affiliations:** 1 Department of Biomedical Research, The Netherlands Organization for Applied Scientific Research (TNO), Leiden, the Netherlands; 2 Department of Cardiology, Leiden University Medical Center (LUMC), Leiden, the Netherlands; 3 Einthoven Laboratory for Experimental Vascular Medicine, LUMC, Leiden, the Netherlands; 4 NewAmsterdam Pharma, Naarden, the Netherlands; 5 Netherlands Heart Institute, Utrecht, the Netherlands; 6 Department of Vascular Medicine, Amsterdam UMC, Amsterdam, the Netherlands; 7 CPC Clinical Research and Division of Cardiology, University of Colorado School of Medicine, Aurora, CO, USA; 8 State University of New York, Downstate School of Public Health, Brooklyn, NY, USA

**Keywords:** atherosclerosis, regression, CETP, non-HDL cholesterol, HDL cholesterol, obicetrapib, ezetimibe

## Abstract

The selective cholesteryl ester transfer protein (CETP) inhibitor obicetrapib is in clinical evaluation for dyslipidemia and cardiovascular risk reduction. This study investigated how obicetrapib alone and with ezetimibe reduces non-HDL-C, affects atherosclerotic lesion progression, and regression when added to background atorvastatin intervention. APOE∗3-Leiden.CETP mice received a Western-type diet (WTD) or this diet supplemented with obicetrapib, ezetimibe, or both. After 8 weeks, all interventions reduced non-HDL-C levels (obicetrapib: −53%; ezetimibe: −19%; combination: −75%). Obicetrapib mono and combination treatment blocked CETP activity (−99% and −98%), thereby increasing HDL-C levels (+286% and +256%). Very low-density lipoprotein (VLDL) cholesterol production was not affected, while obicetrapib and the combination with ezetimibe increased VLDL clearance (plasma half-life [^14^C]-cholesteryloleate: −44% and −57%) and LDL receptor expression (+63% and +74%), without increasing liver lipids. Atherosclerosis progression was evaluated after 28 weeks. All interventions reduced atherosclerotic lesion area (obicetrapib: −90%; ezetimibe: −50%; combination: −98%) and severity due to almost complete reductions in severe lesions (obicetrapib: −80%; combination: −98%). Non-HDL-C exposure (*P* < 0.001) was independently associated with lesion area, in contrast to HDL-C (*P* = 0.336). Combination treatment synergistically reduced non-HDL-C and atherosclerosis. For atherosclerosis regression, mice with advanced and established atherosclerosis received obicetrapib and ezetimibe on top of atorvastatin for 24 weeks. Triple therapy regressed lesion area (−44% vs. baseline) and increased healthy vessel segments, indicating potential for complete atherosclerosis resolution. In summary, obicetrapib, alone or combined with ezetimibe, lowers non-HDL cholesterol by enhancing LDL receptor–mediated VLDL clearance, thereby synergistically reducing atherosclerosis progression, while triple treatment with atorvastatin induces regression of established atherosclerotic lesions.

## Graphical abstract

Obicetrapib alone and in combination with ezetimibe enhance LDL receptor-mediated VLDL clearance and regresses atherosclerosis on atorvastatin background. Treatment with the selective cholesteryl ester transfer protein (CETP) inhibitor obicetrapib combined with ezetimibe results in an increase in high density lipoprotein cholesterol (HDL-C) and apolipoprotein A1 (ApoA1) while concomitantly decreasing non-HDL-C, apolipoprotein B (ApoB) and triglycerides (TG). Furthermore, obicetrapib and ezetimibe decrease plasma proprotein convertase subtilisin kexin type 9 (PCSK9) levels. In the liver, obicetrapib and ezetimibe do not alter very low-density lipoprotein (VLDL) production but increase VLDL clearance as well as LDL receptor protein expression without increasing liver lipid levels. After 28 weeks of treatment, combination treatment with obicetrapib and ezetimibe prevented the formation of atherosclerotic lesions. Moreover, when given on top of atorvastatin, obicetrapib and ezetimibe induced lipid lowering that ultimately resulted in the regression of severe pre-existing atherosclerotic lesions.

**Figure:**
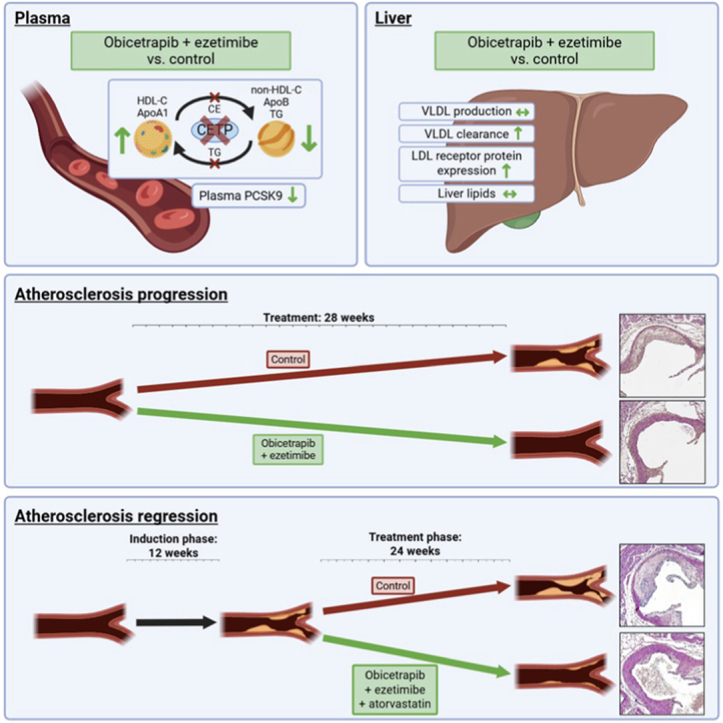

The association between high plasma low-density lipoprotein cholesterol (LDL-C) concentrations and cardiovascular disease (CVD) is well-established. Many patients fail to reach LDL-C goals with conventional therapeutic interventions and therefore remain at risk of developing CVD. While statins are the first-choice pharmacological agents for reducing LDL-C, their benefit in clinical practice remains suboptimal. Therefore, approaches where conventional statin therapy is combined with other pharmacological agents, e.g. ezetimibe or PCSK9 inhibitors, are promoted in accordance with US guidelines (1, 2).

Mendelian randomization studies have predicted the beneficial effects of lower cholesteryl ester transfer protein (CETP) activity on major cardiovascular events (3), and the REVEAL trial with anacetrapib on top of statin intervention showed reduced risk of cardiovascular outcomes (4). However, anacetrapib and other CETP inhibitors, i.e. torcetrapib, dalcetrapib and evacetrapib, failed to reach the clinic due to modest efficacy or adverse side effects (4, 5, 6, 7).

Obicetrapib is a novel oral CETP inhibitor that, in clinical studies, either alone or on top of high-intensity statin intervention, has demonstrated reductions in ApoB and LDL-C levels by up to 30% and 50%, respectively (8, 9, 10). CETP is bound primarily to high-density lipoprotein cholesterol (HDL-C), where it mediates transfer of cholesteryl esters in exchange for triglycerides from HDL to ApoB-containing particles. By inhibiting CETP activity, obicetrapib shifts the plasma lipoprotein profile towards more HDL-C particles and fewer atherogenic (very) low-density lipoprotein cholesterol ((V)LDL-C) particles. Ezetimibe decreases intestinal absorption of cholesterol (11), thereby reducing circulating LDL-C levels in humans by about 15%–25%, and improving cardiovascular outcomes when added to background statin therapy (12). Since obicetrapib and ezetimibe have complementary targets, their combination has been shown to provide additional improvements in atherogenic lipid profiles (13), which is beneficial for patients who fail to achieve their LDL-C targets with conventional statin intervention. However, the precise mechanisms through which obicetrapib and ezetimibe improve atherogenic lipid profiles remain incompletely understood.

To investigate the effects of combination treatment on both mechanism of action and atherosclerosis progression and regression, APOE∗3-Leiden.CETP mice were used. APOE∗3-Leiden mice possess a delayed but functional ApoE-LDLR-mediated clearance pathway (14, 15). Introduction of the human CETP gene has resulted in a model with human-like lipoprotein metabolism (16, 17). Moreover, APOE∗3-Leiden.CETP mice respond well to lipid-lowering interventions including those used in the clinic, like statins (18, 19, 20, 21), ezetimibe (19), PCSK9 inhibitors (20, 22, 23), ANGPTL3 inhibitors (21, 24, 25), HDL-raising treatments (26, 27, 28), and CETP inhibitors (29, 30, 31). In fact, using this model, we predicted that the reduced risk of cardiovascular endpoints with anacetrapib in the REVEAL trial can be ascribed to its non-HDL-C lowering effect and not to its HDL-C raising effect, which was subsequently confirmed in the REVEAL study (4, 29). To mimic human dietary fat and cholesterol intake, mice received a Western-type diet (WTD) with low cholesterol content (equivalent to daily human intake) to induce atherosclerosis development. This study aimed to elucidate the mechanism underlying the reduction in non-HDL-C and to assess the effects of obicetrapib and ezetimibe on atherosclerosis development, as well as their effects on lesion regression when given on top of atorvastatin.

## Materials and Methods

All experimental information is outlined in full detail in the Supplementary Material.

### Ethics and experimental design

Animal care and experimental procedures were approved by The Netherlands Central Authority for Scientific Procedures on Animals (CCD; project license: AVD5010020172064). This study was approved by an independent Animal Welfare Body of TNO (IvD TNO; TNO-526/532/555/565). APOE∗3-Leiden.CETP transgenic mice were bred and housed at the AAALAC-accredited SPF animal facility at TNO (TNO Metabolic Health Research, Leiden, the Netherlands). A schematic overview of the study set-up is given in Supplementary Fig. S1.

### Statistical analysis

Statistical analyses were performed using SPSS software (version 25, IBM Corp) or SAS software (version 9.4, SAS institute). Differences between groups were determined non-parametrically by Kruskal-Wallis testing followed by Mann-Whitney U testing for independent samples. For metabolic parameters, a Bonferroni-Holm correction was performed to correct for multiple testing. All treatment groups were compared with the control group and both monotreatment groups were compared with the combination treatment group.

The Bliss definition of independence (32) predicts the combined effect size of treatment A (obicetrapib) and treatment B (ezetimibe) under the assumption that their effects are independent. Accordingly, the expected effect of combination treatment is predicted as: Effect_AB_ = Effect_A_ + Effect_B_ – (Effect_A_ × Effect_B_). A synergistic effect is present when the observed effect of the combination exceeds the predicted effect based on Bliss independence, indicating that the treatments interact in a way that enhances their combined efficacy. More details on the statistical analyses performed in this study, including on the contribution of HDL-C and non-HDL-C to atherosclerosis development, assumptions for the Bliss definition of independence and contribution of HDL-C and non-HDL-C lowering to atherosclerosis regression are given in the Supplementary Material.

## Results

### Obicetrapib monotreatment and the combination with ezetimibe strongly reduce ApoB and non-HDL-C while increasing HDL-C

**Fig. 1:**
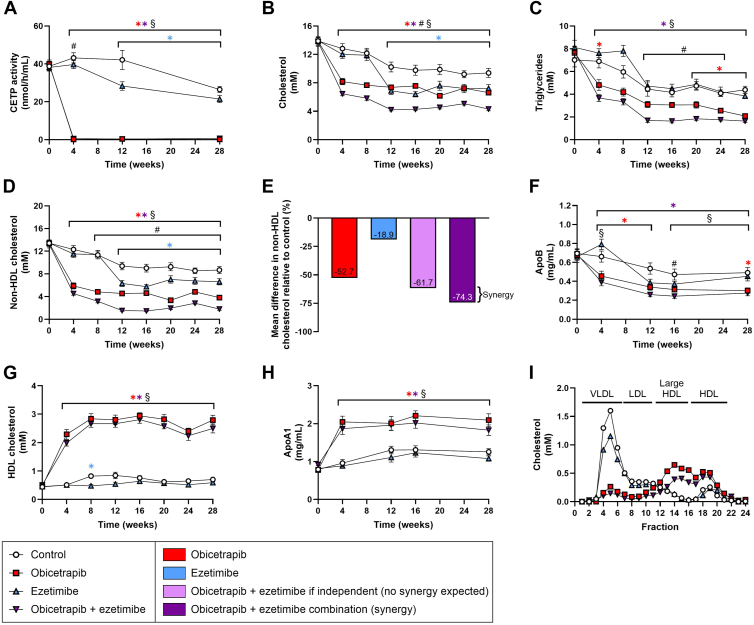
Obicetrapib, ezetimibe and the combination of obicetrapib and ezetimibe improve plasma lipids. CETP activity (A), total cholesterol (B), triglycerides (C) and non-HDL-C (D). Differences in non-HDL-C relative to control (E) where bars represent mean percent differences in non-HDL-C for obicetrapib (first bar from the left), ezetimibe (second bar from the left), and the results observed for the combination (rightmost bar). The observed mean percent difference for the combination (−74.3%) was greater than what would be expected if the compounds had independent effects (−61.7%, two bars from the right) suggesting a synergistic effect of obicetrapib and ezetimibe. Plasma ApoB (F), HDL-C (G), ApoA1 (H) and lipoprotein profiles for cholesterol (I) were determined throughout the study or at the study endpoint. Data are presented as mean ± SEM (n = 10 per group). ∗*P* < 0.05 versus control, #*P* < 0.05 obicetrapib versus obicetrapib + ezetimibe, §*P* < 0.05 ezetimibe versus obicetrapib + ezetimibe.

To evaluate the effects of obicetrapib and ezetimibe on atherogenic lipids, mice were treated for 28 weeks while on a WTD with 0.05% cholesterol. Obicetrapib almost completely blocked CETP activity (obicetrapib: −99%; combination: −98%, vs. control at endpoint) (Fig. 1A). Relative to control mice, ezetimibe reduced CETP activity at the study endpoint as well (−19%). Total cholesterol levels were significantly decreased by obicetrapib monotreatment at all timepoints compared to control mice (on average −31%) (Fig. 1B). In the groups treated with ezetimibe, dosing was adjusted at several timepoints to reduce total cholesterol concentrations to reach similar cholesterol-lowering as observed in patients, resulting in an average reduction of 14% over the total intervention period. Combination of obicetrapib and ezetimibe further reduced plasma cholesterol (−43% vs. control). Obicetrapib significantly reduced plasma triglycerides throughout the study (on average −34% vs. control), whereas triglycerides were not altered by ezetimibe compared to control (Fig. 1C). The combination of obicetrapib and ezetimibe further reduced average triglyceride levels, both relative to control (−55%) and obicetrapib alone (−32%).

The cholesterol-lowering effects of obicetrapib and ezetimibe could be attributed to reduced average non-HDL-C levels (obicetrapib: −53%; ezetimibe: −19%; combination: −75%, vs. control) (Fig. 1D). To establish whether treatment with obicetrapib and ezetimibe had a synergistic or additive effect on non-HDL-C lowering, the Bliss independence model was used to test the relation of their interaction. The observed mean percentage of difference in non-HDL-C relative to control for the combination (−74.3%) was greater than what would be expected if the compounds had independent effects (−61.7%). This suggests a statistically significant synergistic effect of 48.9% (p_interaction_ = 0.0005) of obicetrapib and ezetimibe in reducing non-HDL-C levels (Fig. 1E).

Consistent with non-HDL-C lowering, obicetrapib alone and with ezetimibe significantly reduced ApoB concentrations (−27% and −35%, respectively) while ezetimibe alone did not affect ApoB (Fig. 1F). Additionally, obicetrapib significantly increased plasma HDL-C levels (obicetrapib: 2.9-fold increase; combination: 2.6-fold increase, vs. control at endpoint), while HDL-C was not affected by ezetimibe (Fig. 1G). Similarly, average plasma ApoA1 concentrations, a major component of HDL particles, were significantly higher in mice treated with obicetrapib alone (+63%) and in combination with ezetimibe (+53%) (Fig. 1H). In untreated control mice, cholesterol was mainly confined to (V)LDL particles and to a lesser extent to HDL particles, a pattern also observed in ezetimibe-treated mice (Fig. 1I). Mice that received obicetrapib alone or in combination with ezetimibe showed a shift in lipoprotein profile, with minimal cholesterol confined to (V)LDL and instead formation of (large) HDL particles.

### Obicetrapib monotreatment and the combination with ezetimibe enhance VLDL clearance from the circulation

To clarify how obicetrapib and ezetimibe reduce non-HDL-C, separate studies were conducted to determine VLDL production and clearance rates. For measurement of VLDL production, lipoprotein lipase (LPL)-mediated lipolysis and consequently VLDL clearance were completely blocked via Triton WR-1339 administration, after which plasma triglyceride concentrations were determined in the following 60 min. Neither of the treatments affected the increase in plasma triglycerides (supplementary Fig. S2A) and consequently total VLDL-TG production was similar between all groups (supplementary Fig. S2B). Correspondingly, there were no differences in de novo VLDL-ApoB production rate (supplementary Fig. S2C). These findings indicate that the reductions in non-HDL-C induced by obicetrapib, ezetimibe and their combination are not the result of reduced VLDL production.

**Fig. 2:**
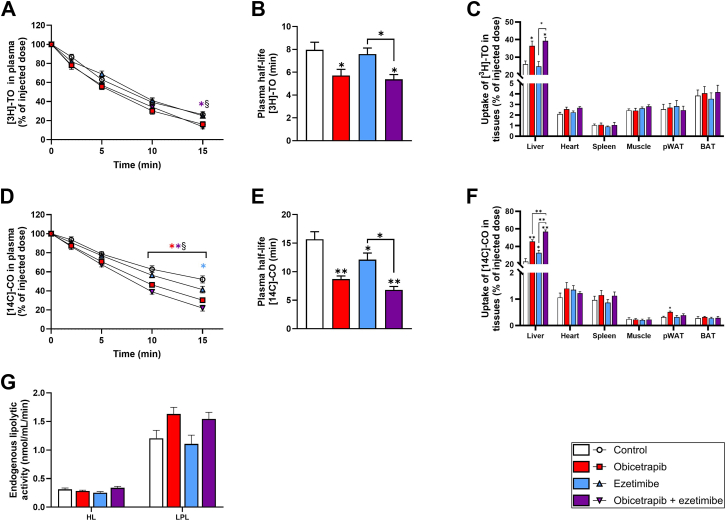
VLDL clearance is increased in mice treated with obicetrapib alone and in combination with ezetimibe. Mice were injected with VLDL-like particles containing glycerol tri[^3^H]oleate ([^3^H]-TO) and [^14^C]-cholesteryloleate ([^14^C]-CO). [^3^H]-TO plasma decay is plotted in (A) and was used to calculate the plasma half-life of these particles (B). Clearance of [^3^H]-TO particles in different organs was determined (C). [^14^C]-CO plasma decay is plotted in (D) and was used to calculate the plasma half-life of these particles (E). Clearance of [^14^C]-CO particles in different organs was determined (F). Endogenous lipolytic activity was determined by measuring hepatic lipase (HL) and lipoprotein lipase (LPL) activity (G). Data are presented as mean ± SEM (n = 10 per group). ∗*P* < 0.05, ∗∗*P* < 0.01 versus control or indicated treatments, #*P* < 0.05 obicetrapib versus combination, §*P* < 0.05 ezetimibe versus combination. BAT, brown adipose tissue; pWAT, perigonadal white adipose tissue.

To investigate whether the reduction in non-HDL-C by obicetrapib and ezetimibe was caused by increased VLDL clearance from the circulation, double-radiolabeled VLDL-mimicking particles were injected intravenously, and their clearance from plasma and uptake in different tissues were measured. The uptake of glycerol tri[^3^H]oleate ([^3^H]-TO), representing free fatty acids and triglycerides, was faster in mice treated with obicetrapib monotreatment and the combination with ezetimibe compared to untreated control mice and mice treated with ezetimibe alone (Fig. 2A). The plasma half-life of these particles was significantly lower in mice treated with obicetrapib alone (−28%) and in combination with ezetimibe (−33%) (Fig. 2B), indicating an increased clearance rate from the circulation. In these mice, [^3^H]-TO particles were primarily taken up by the liver (obicetrapib: +40%; combination: +51%) (Fig. 2C). The clearance rate of [^14^C]-cholesteryl oleate ([^14^C]-CO) VLDL-mimicking particles, representing cholesterol, was even more pronounced, with all interventions showing an increased clearance 15 min post-injection (Fig. 2D). This resulted in a significantly reduced plasma half-life of these particles (obicetrapib: −44%; ezetimibe: −23%; combination: −57%) (Fig. 2E). Obicetrapib and ezetimibe significantly increased liver uptake of [^14^C]-CO particles as compared to control (obicetrapib: +99%; ezetimibe: +43%) (Fig. 2F). Combination of obicetrapib and ezetimibe further increased liver uptake of [^14^C]-CO particles, both relative to untreated control mice (2.5-fold increase), obicetrapib alone (+24%) and ezetimibe alone (+73%).

Lipolytic activity, assessed by measuring post-heparin activity of hepatic lipase (HL) and LPL (Fig. 2G), did not significantly differ between control and treated animals. Collectively, these data indicate that the lipid-lowering effect of obicetrapib monotreatment and the combination with ezetimibe is mediated through increased hepatic uptake of VLDL remnant particles and may be independent from lipase-mediated hydrolysis of VLDL-triglycerides.

### Obicetrapib monotreatment and the combination with ezetimibe decrease plasma PCSK9 levels and increase hepatic LDL receptor expression without increasing hepatic lipids

**Fig. 3:**
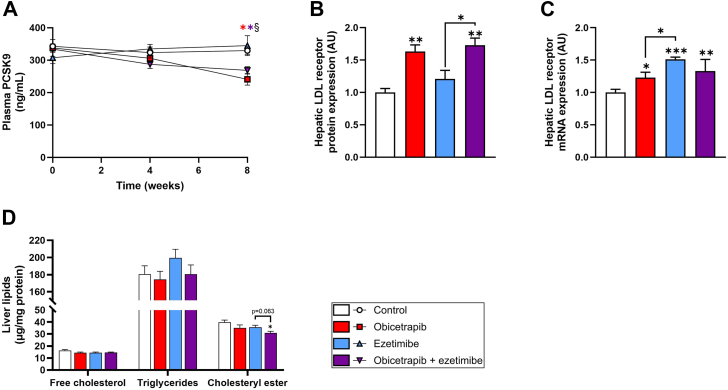
Obicetrapib decreases plasma PCSK9, resulting in increased hepatic LDL receptor protein expression. Plasma PCSK9 concentrations (A), hepatic LDL receptor protein expression (B), hepatic LDL receptor mRNA expression (C), and hepatic concentrations of free cholesterol, triglycerides and cholesteryl ester (D). Data are presented as mean ± SEM (n = 10 per group). ∗*P* < 0.05, ∗∗*P* < 0.01, ∗∗∗*P* < 0.001 versus control or indicated treatments, §*P* < 0.05 ezetimibe versus combination.

To further investigate the underlying mechanism for the increased VLDL clearance from the circulation, plasma proprotein convertase subtilisin kexin type 9 (PCSK9) concentrations and hepatic LDL receptor levels were determined. LDL receptor-mediated hepatic uptake of ApoB-containing lipoproteins is the most important pathway for removal of these atherogenic particles from the circulation. PCSK9 is a major determinant of LDL receptor expression on the hepatocyte membrane, thereby mediating LDL-C endocytosis (33). Plasma PCSK9 levels were significantly reduced by obicetrapib alone (−27%) and in combination with ezetimibe (−18%) (Fig. 3A). Consistent with the decrease in circulatory PCSK9 concentrations, hepatic LDL receptor expression was increased in mice treated with obicetrapib alone (+63%) and in combination with ezetimibe (+74%) (Fig. 3B). In addition, LDLR mRNA expression increased following CETP inhibition by obicetrapib (Fig. 3C), indicating that the transcriptional upregulation by obicetrapib contributes to the elevated LDLR protein levels. We also measured hepatic protein levels (data no shown) of LRP1 (no differences between group) and VLDLR (not detectable), indicating that the observed reductions in non-HDL-C are driven primarily by increased LDL receptor-mediated clearance, rather than compensatory upregulation of LRP1 and VLDLR.

The increased VLDL clearance may result in accumulation of lipids in the liver and therefore hepatic lipid contents were evaluated. Liver free cholesterol and triglycerides remained unchanged with either obicetrapib or ezetimibe (Fig. 3D). Hepatic cholesteryl esters, on the other hand, were significantly reduced in the combination treatment group compared to untreated control mice (−22%) and showed a trend towards a significant reduction compared to mice treated with ezetimibe alone (−13%, *P* = 0.063) (Fig. 3D). These data indicate that the uptake of lipoprotein remnants is more efficient in livers of mice treated with obicetrapib and ezetimibe compared to untreated control mice, and do not result in increased hepatic lipid levels.

### Obicetrapib monotreatment and the combination with ezetimibe strongly reduce atherosclerosis progression to a greater extent than ezetimibe alone

**Fig. 4:**
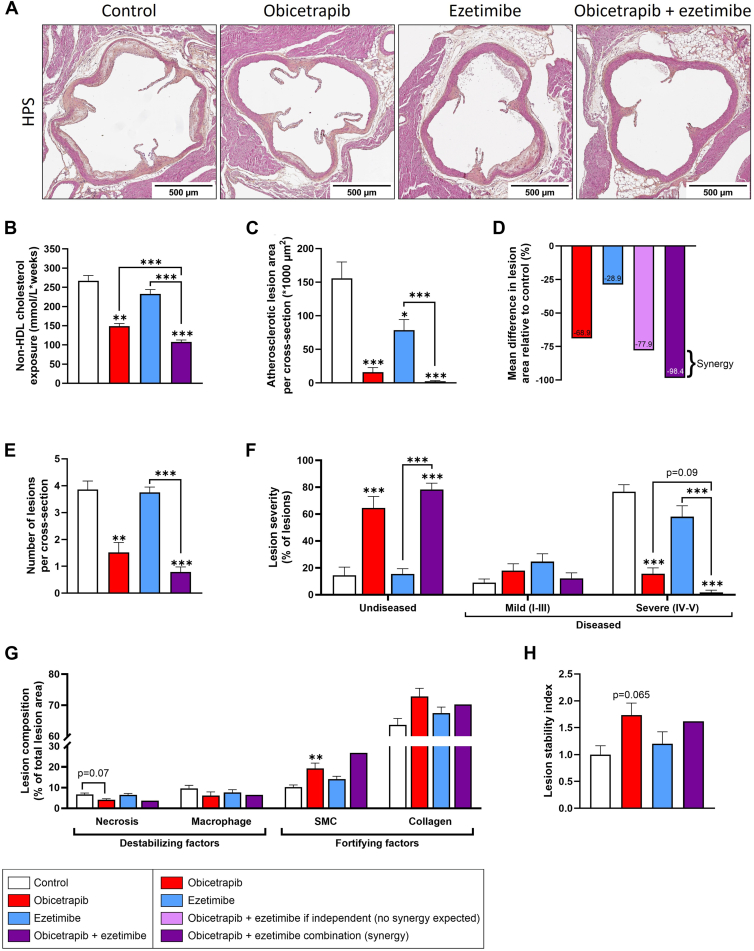
Progression of atherosclerosis is strongly reduced by obicetrapib, ezetimibe and the combination thereof. Atherosclerosis was assessed in hematoxylin-phloxine-saffron (HPS)-stained cross-sections of the aortic root area (A). Total non-HDL-C exposure (concentration × weeks) (B) and atherosclerotic lesion area (C). Differences in atherosclerotic lesion area relative to control where bars represent mean percent differences in lesion area, adjusted for baseline non-HDL-C, for obicetrapib (first bar from the left), ezetimibe (second bar from the left), and the results observed for the combination (rightmost bar). The observed mean percent difference for the combination (−98.4%) was greater than what would be expected if the compounds had independent effects (−77.9%, two bars from the right), suggesting a synergistic effect of obicetrapib and ezetimibe (D). Number of atherosclerotic lesions (E), atherosclerotic lesion severity (F). Complex (type IV-V) lesions were analyzed for necrotic core and macrophage content (destabilizing factors) and smooth muscle cell (SMC) and collagen content (fortifying factors) (G) and lesion stability index was calculated by dividing the sum of plaque stabilizing factors by the sum of plaque destabilizing factors (H). Data are presented as mean ± SEM (n = 14–15 per group). ∗*P* < 0.05, ∗∗*P* < 0.01, ∗∗∗*P* < 0.001 versus control or indicated treatments.

The lipid-lowering effects of obicetrapib, presented in Figure 1, resulted in significantly lower non-HDL-C exposure (defined as plasma levels × weeks) throughout the 28-week intervention period compared to control mice (obicetrapib: −44%) (Fig. 4B). Combination of obicetrapib with ezetimibe reduced non-HDL-C exposure even further (−60% vs. control; −28% vs. obicetrapib monotreatment). Consequently, atherosclerotic lesion area was significantly reduced, with almost no lesions in the combination intervention group (obicetrapib: −90%; ezetimibe: −50%; combination: −98%, vs. control) (Fig. 4A + C). The observed mean percentage difference in lesion area relative to control for the combination (−98.4%) was greater than what would be expected if the compounds had independent effects (−77.9%) (p_interaction_ between obicetrapib and ezetimibe was 0.0356), suggesting a synergistic effect of obicetrapib and ezetimibe in reducing atherosclerotic lesion area (Fig. 4D).

Obicetrapib, either alone or with ezetimibe, significantly reduced the number of atherosclerotic lesions (obicetrapib: −61%; combination: −80%, vs. control), an effect not observed in ezetimibe-treated mice (Fig. 4E). Subsequent evaluation of lesion severity revealed a significant increase in the percentage of undiseased segments (obicetrapib 4.5-fold increase; combination: 5.4-fold increase, vs. control) (Fig. 4F). Additionally, in mice treated with obicetrapib alone, there was limited progression towards more advanced stages, with fewer severe (type IV-V) lesions compared to control mice (−80%) (Fig. 4F). This effect was even stronger in the combination treatment group, where formation of severe lesions was almost completely absent (−98%).

Further analysis of the vulnerable (34, 35, 36) severe (type IV-V) lesions was done by evaluating necrotic core and macrophage content (destabilizing factors) as well as collagen and smooth muscle cell (SMC) content (stabilizing factors) (Supplementary Fig. S3). It is important to note that for this analysis, only type IV and V lesions were analyzed. In the combination treatment group, where atherosclerosis was nearly absent, only one mouse presented with three severe lesions, which prevented statistical analysis for this group.

Obicetrapib tended to reduce necrotic core content (−39%), while macrophage levels remained unchanged across all groups (Fig. 4G). Among plaque-stabilising factors, obicetrapib significantly increased SMC content (+88%), whereas ezetimibe had no effect. SMC content also appeared elevated in the combination treatment group (one mouse). Collagen content was not affected by either treatment. Plaque stability index was calculated by dividing stabilizing factors (collagen + SMC content) by destabilizing factors (macrophage + necrotic core content) and showed a borderline significant increase in obicetrapib-treated mice (+74%, *P* = 0.065) that was not observed in ezetimibe-treated mice (Fig. 4H).

### Obicetrapib reduces atherosclerosis progression primarily by reducing non-HDL-C exposure

**Fig. 5:**
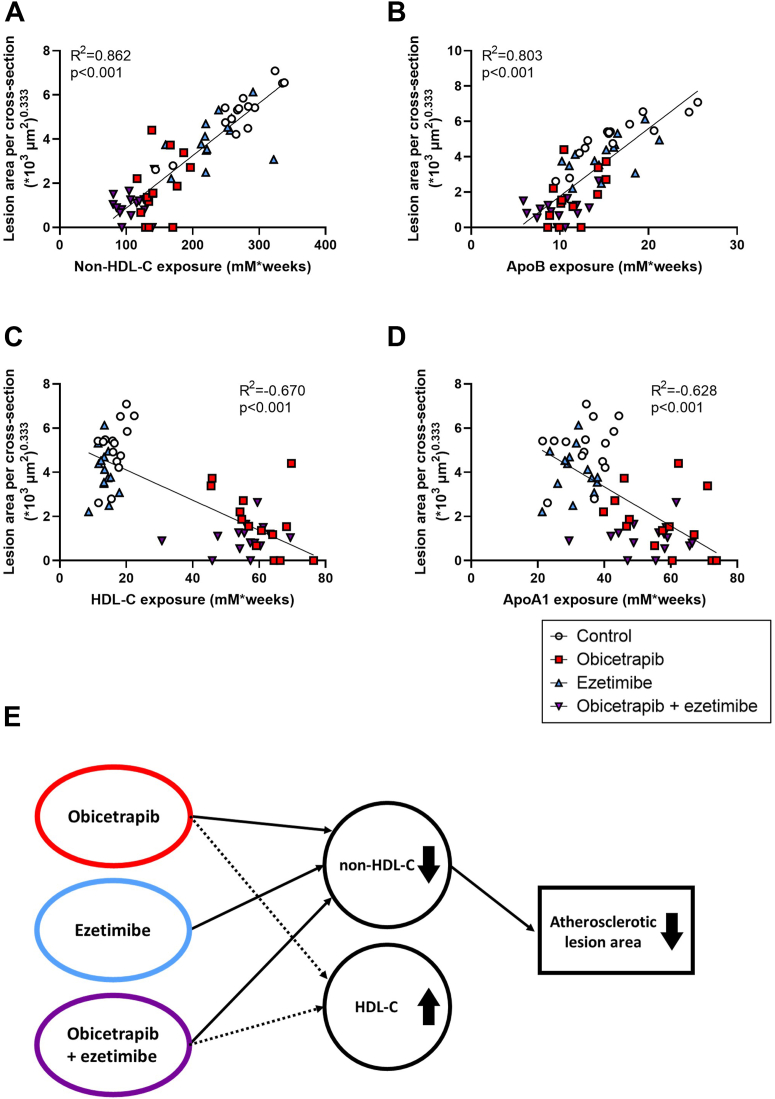
Correlations between plasma lipids and atherosclerotic lesion area. Linear regression analyses were performed by plotting the cubic root of atherosclerotic lesion area against non-HDL-C exposure (A), ApoB exposure (B), HDL-C exposure (C) or ApoA1 exposure (D). Hypothetical scheme of factors contributing to atherosclerotic lesion area as suggested by statistical analyses (E). To this end, analysis of covariance (ANCOVA) was performed to test for group differences in atherosclerotic lesion area with HDL-C exposure and non-HDL-C exposure as covariates. Non-HDL-C exposure but not HDL-C exposure independently determined atherosclerotic lesion size. The effects of obicetrapib and ezetimibe on atherosclerosis development were mainly mediated through reductions in non-HDL-C exposure.

To evaluate whether the effects of obicetrapib and ezetimibe on atherosclerosis development could be explained by the increase in HDL-C, the decrease in non-HDL-C or both, a univariate regression analysis was performed. This univariate analysis showed that atherosclerotic lesion area was strongly predicted by non-HDL-C exposure (*R*^2^ = 0.86, *P* < 0.001) (Fig. 5A) and ApoB exposure (*R*^2^ = 0.80, *P* < 0.001) (Fig. 5B) and to a lesser degree by total HDL-C exposure (*R*^2^ = −0.67, *P* < 0.001) (Fig. 5C) and ApoA1 exposure (*R*^2^ = −0.63, *P* < 0.001) (Fig. 5D).

An analysis of covariance (ANCOVA) was performed and showed that non-HDL-C (*P* < 0.001) but not HDL-C (*P* = 0.336) independently determined atherosclerotic lesion size. To assess the degree of correlation between HDL-C and non-HDL-C, the variance inflation factors (VIF = 2.426) and condition index (CI = 2.708) were calculated. Both values did not exceed the threshold for collinearity, indicating that collinearity between HDL-C and non-HDL-C is unlikely to pose a significant concern in the regression model. These analyses demonstrate that the ameliorative effect of obicetrapib alone and in combination with ezetimibe on atherosclerosis can be explained mainly by the reduction in non-HDL-C and not by the increase in HDL-C (Fig. 5E).

### Double and triple treatment with obicetrapib and ezetimibe on top of atorvastatin reduces TC and non-HDL-C

We have previously demonstrated that high-intensity, cholesterol-lowering triple treatment with atorvastatin, alirocumab, and evinacumab in APOE∗3-Leiden.CETP mice regresses pre-existent atherosclerotic lesions (21). To study whether obicetrapib and ezetimibe treatment on top of atorvastatin causes regression of atherosclerosis, mice were fed a WTD with 0.3% cholesterol for 12 weeks to induce severe hyperlipidemia and consequently atherosclerosis. After this induction period, at t = 0 weeks, mice were either sacrificed (baseline control) or switched to the WTD with 0.05% cholesterol without intervention (control) or with atorvastatin, obicetrapib and ezetimibe or the combination of obicetrapib, ezetimibe and atorvastatin for another 24 weeks.

**Fig. 6:**
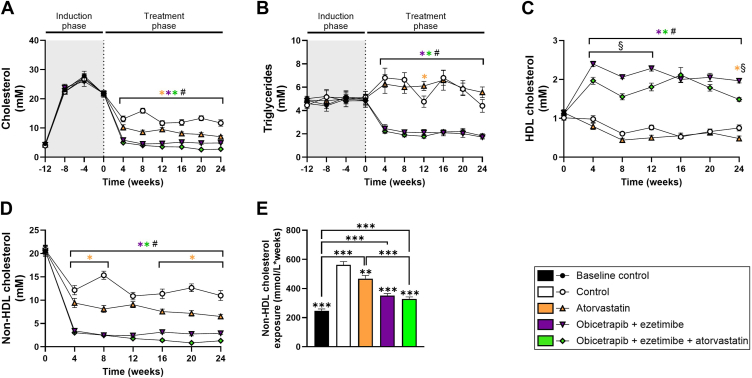
Obicetrapib and ezetimibe on top of atorvastatin improve plasma lipids. Total cholesterol (A), triglycerides (B), HDL cholesterol (C), non-HDL cholesterol (D) and total non-HDL cholesterol exposure (concentration × weeks) (E). Data are presented as mean ± SEM (n = 15 per group). ∗*P* < 0.05, ∗∗*P* < 0.01, ∗∗∗*P* < 0.001 versus control or indicated treatments, #*P* < 0.05 atorvastatin versus triple intervention (obicetrapib, ezetimibe, atorvastatin), §*P* < 0.05 double intervention (obicetrapib, ezetimibe) versus triple intervention (obicetrapib, ezetimibe, atorvastatin).

Plasma cholesterol levels were decreased in all treatment groups (on average atorvastatin: −34%; obicetrapib + ezetimibe: −61%; obicetrapib + ezetimibe + atorvastatin: −72% vs. control) (Fig. 6A). Average plasma triglycerides were not affected by atorvastatin alone, while triglycerides were significantly lowered in the other treatment groups relative to control mice at all timepoints (obicetrapib + ezetimibe: −64%; obicetrapib + ezetimibe + atorvastatin: −66%) (Fig. 6B). HDL-C levels remained similar in atorvastatin-treated mice, while their levels were elevated in mice treated with obicetrapib and ezetimibe (3.0-fold increase) and mice treated with obicetrapib, ezetimibe and atorvastatin (2.5-fold increase), relative to untreated controls (Fig. 6C). Average plasma non-HDL-C concentrations were significantly reduced in all treatment groups versus untreated control mice (atorvastatin: −35%; obicetrapib + ezetimibe: −77%; obicetrapib + ezetimibe + atorvastatin: −85%) (Fig. 6D). Triple treatment showed the largest reduction with non-HDL-C levels down to 1.3 mmol/L during the last 4 weeks of the intervention. The total non-HDL-C exposure (concentration × weeks) was significantly lowered by atorvastatin (−17%), by obicetrapib and ezetimibe (−38%) and to the largest extent by the triple treatment group (−42% vs. control), which was significantly lower when compared to atorvastatin monotreatment (−30%) (Fig. 6E). These data demonstrate that double treatment with obicetrapib and ezetimibe, as well as triple therapy including atorvastatin, dramatically reduces plasma concentrations of atherogenic lipids.

### Triple treatment with obicetrapib and ezetimibe on top of atorvastatin regresses pre-existent atherosclerosis

**Fig. 7:**
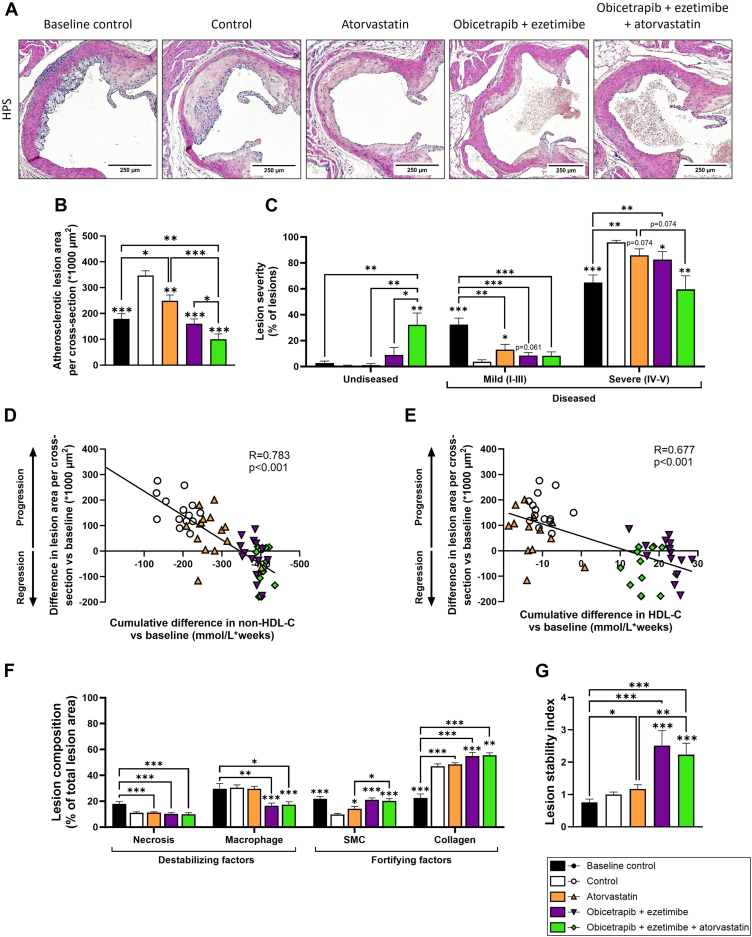
Triple treatment with obicetrapib and ezetimibe on top of atorvastatin regresses pre-existent atherosclerotic lesions. Atherosclerosis was assessed in hematoxylin-phloxine-saffron (HPS)-stained cross-sections of the aortic root area (A). Atherosclerotic lesion area per cross-section (B) and lesion severity (C). Correlation between the cumulative decrease in plasma non-HDL-C and atherosclerotic lesion area (D). Mean non-HDL-C levels at baseline were subtracted from non-HDL-C levels of each individual mouse at each timepoint and cumulative decrease in non-HDL-C exposure during treatment was calculated as concentration × weeks. Data were plotted against the mean difference in lesion area at the study endpoint and mean lesion area at baseline. Linear regression analysis was performed (n = 15 per group). Similarly, correlation between the cumulative increase in HDL-C levels and atherosclerotic lesion area was calculated (E). Complex (type IV-V) lesions were analyzed for necrotic core and macrophage content (destabilizing factors) and smooth muscle cell (SMC) and collagen content (fortifying factors) (F) and lesion stability index was calculated by dividing the sum of plaque stabilizing factors by the sum of plaque destabilizing factors (G). Data are presented as mean ± SEM (n = 15 per group). ∗*P* < 0.05, ∗∗*P* < 0.01, ∗∗∗*P* < 0.001 versus control or indicated treatments.

Finally, we assessed the effect of intensive lipid lowering by double and triple treatment on the regression of pre-existing atherosclerotic lesions in the aortic root (Fig. 7A). Twelve weeks of WTD with 0.3% cholesterol feeding induced development of advanced atherosclerotic lesions (baseline control; Fig. 7A, B). An additional 24 weeks on WTD with 0.05% cholesterol further progressed atherosclerosis by +94% (control; Fig. 7A, B). In the control group, almost all lesions were severe type IV and V lesions (Fig. 7C). Atorvastatin monotreatment modestly reduced atherosclerosis progression (−28%), slightly shifting lesion severity to more mild and fewer severe lesions. Double treatment with obicetrapib and ezetimibe fully blocked further progression of atherosclerosis, with a similar atherosclerotic lesion area compared to baseline control. Triple treatment with obicetrapib, ezetimibe and atorvastatin not only blocked progression (−71% vs. control) but resulted in regression of pre-existent lesions (−44%) compared to baseline control (Fig. 7B). Triple treatment resulted in a significant reduction in severe lesions (−38%) compared to control and a significant reduction in mild lesions compared to baseline control (−75%). Notably, undiseased segments were virtually absent in both the baseline control and control group, whereas in the triple treatment group, a third of all segments (32%) were undiseased. These findings indicate that triple treatment with obicetrapib, ezetimibe and atorvastatin not only halts the progression of severe atherosclerotic lesions and promotes their regression but also results in the restoration of vascular health, showing the potential for complete resolution of atherosclerosis.

We evaluated whether the reduction in atherosclerotic lesion area could be explained by the reduction in plasma non-HDL-C levels and the increase in HDL-C levels during the intervention period. To this end, the mean non-HDL-C level at baseline (t = 0) was subtracted from the non-HDL-C levels of each individual mouse and at each time point. The cumulative decrease in non-HDL-C exposure was calculated (concentration × weeks) from the start of the intervention period until the study endpoint. These data were plotted against the difference in atherosclerotic lesion area versus baseline (Fig. 7D). Univariate regression analysis revealed a strong significant correlation (R = 0.78) between the difference in lesion area and cumulative decrease in non-HDL-C. Similarly, univariate regression analysis showed a significant correlation between increased HDL-C levels and decreased atherosclerotic lesion area (R = 0.677) (Fig. 7E). These findings highlight the effects of therapeutic non-HDL-C lowering and parallel HDL-C raising on the regression of atherosclerotic lesions.

We further analyzed the vulnerable (34, 35, 36) severe (type IV-V) lesions by evaluating necrotic core and macrophage content (destabilizing factors) as well as collagen and smooth muscle cell (SMC) content (stabilizing factors) (Supplementary Fig. S4). Necrotic core content was unchanged in all treatment groups compared to the control group (Fig. 7F). In the double and triple treatment groups, macrophage content was significantly reduced compared to the control group (−46% and −43%, respectively) (Fig. 7F). Compared to the control group, SMC content was significantly higher in all intervention groups (atorvastatin: +45%; obicetrapib + ezetimibe: +115%; obicetrapib + ezetimibe + atorvastatin: +107%) (Fig. 7F). While atorvastatin alone did not alter collagen content in the plaque, double and triple treatment increased collagen content relative to controls (+17% and +18%, respectively) (Fig. 7F). Plaque stability index, calculated as the sum of lesion stabilizing factors (collagen + SMC content) by lesion destabilizing factors (macrophage + necrotic core content), showed no change in the atorvastatin-treated group versus control (Fig. 7G). However, mice treated with obicetrapib and ezetimibe and mice treated with obicetrapib, ezetimibe, and atorvastatin had significantly more stable atherosclerotic lesions compared to untreated controls (+151% and +123%) (Fig. 7G).

## Discussion

The CETP inhibitor obicetrapib has been shown to substantially improve lipid and lipoprotein profiles in dyslipidemic patients, either alone or in combination with ezetimibe (8, 9, 10, 37). Using a mouse model of hyperlipidemia and atherosclerosis under dietary fat and cholesterol conditions that mimic daily human intake, we evaluated the mechanism underlying the reduction in non-HDL-C and assessed the effects of obicetrapib and ezetimibe on atherosclerosis progression and regression. Our data reveal that obicetrapib and ezetimibe reduce non-HDL-C levels by enhancing LDL receptor-mediated VLDL clearance, thereby synergistically inhibiting atherosclerosis progression. Additionally, when given on top of atorvastatin, obicetrapib and ezetimibe induce lipid-lowering that ultimately results in the regression of severe pre-existent lesions as well.

While currently available therapies aim mostly to decrease plasma LDL-C, remnant cholesterol and triglycerides are considered to be equally important residual risk factors for CVD (38, 39), and non-HDL-C and ApoB appear to be superior to LDL-C in CVD risk estimation (40). The clinical benefit of lowering triglycerides and LDL-C may be proportional to the absolute change in ApoB, implicating that all ApoB-containing lipoproteins have approximately the same effect on CVD risk per particle (41). In this study, we demonstrate that non-HDL-C levels are particularly predictive for the development of atherosclerosis in an established animal model for hyperlipidemia and atherosclerosis.

In the present study in APOE∗3-Leiden.CETP mice, obicetrapib completely blocked CETP activity, resulting in an average 53% reduction in non-HDL-C and 290% increase in HDL-C concentrations. This is in line with clinical observations in phase 1 and 2 studies using obicetrapib, demonstrating LDL-C lowering of up to 51% and HDL-C increases up to 179% (8, 9, 10, 37). In combination with ezetimibe, non-HDL-C concentrations were lowered even further, by on average 74% relative to control. This synergistic effect of ezetimibe is consistent with changes observed in patients with elevated LDL-C who were treated with the combination of obicetrapib and ezetimibe, showing a 63% reduction (37). Here, obicetrapib shifted lipoprotein profiles to a phenotype where cholesterol was minimally confined in (V)LDL particles. Consistent with clinical data (37), obicetrapib alone or in combination with ezetimibe induced the development of large HDL particles and our findings do not indicate any negative impact of these large HDL particles on atherosclerosis. On the contrary, our data show that HDL contributed to regression of atherosclerosis.

Similar to non-HDL-C concentrations, intervention with obicetrapib in the APOE∗3-Leiden.CETP mouse model resulted in a 27% reduction in plasma ApoB concentrations, which were lowered even further in combination with ezetimibe, by 35%. In clinical studies, obicetrapib intervention was shown to lower ApoB levels as well, by up to 40% (8, 9, 37). In the ROSE2 clinical study in dyslipidemic patients, obicetrapib alone lowered ApoB levels by 24% and in combination with ezetimibe by 34% (37). In line with the rise in HDL-C concentrations in APOE∗3-Leiden.CETP mice, obicetrapib intervention induced an increase in ApoA1 levels by 63% and in combination with ezetimibe by 53%. These data are consistent with clinical studies, where obicetrapib induced ApoA1 elevations up to 63% (8, 9).

The beneficial effects of obicetrapib and ezetimibe on plasma lipids could not be attributed to a reduction in VLDL production but rather to an increased VLDL clearance from the circulation. In the current study, we have demonstrated that obicetrapib combined with ezetimibe increases clearance of labeled VLDL-like particles, which are primarily taken up by the liver. Although not statistically significant, LPL expression showed an upward trend in obicetrapib-treated groups, consistent with the observed improvements in plasma lipid clearance. This increased clearance, in turn, was caused by a decrease in plasma PCSK9 levels and an increase in hepatic LDL receptor expression, allowing for more efficient uptake of lipoprotein remnants in mice treated with both obicetrapib and ezetimibe. The data suggest that the decreases in atherogenic lipids and lipoproteins previously described in the clinic are secondary to increased hepatic clearance without increasing hepatic lipid content.

As a consequence of the reduced exposure to non-HDL-C and ApoB, atherosclerotic lesion progression was strongly inhibited in mice treated with obicetrapib alone or in combination with ezetimibe, reflected by the reductions in atherosclerotic lesion area as well as the number of lesions per cross-section. Obicetrapib monotreatment shifted lesion severity toward a phenotype with more undiseased and fewer severe lesions. The anti-atherogenic effects were strongest in the combination treatment group, where atherosclerosis development was minimal, and lesions that did develop were mostly mild in nature. Furthermore, severe (type IV-V) atherosclerotic lesions tended to be more stable in mice treated with obicetrapib. Correlation analysis revealed that the ameliorative effects of obicetrapib and ezetimibe on atherosclerosis progression could primarily be explained by the reduction in non-HDL-C, whereas HDL-C was not an independent predictor of the lesion area when non-HDL-C was included as covariate.

Subsequently, the effects of obicetrapib and ezetimibe on top of atorvastatin were evaluated on the regression of pre-existent atherosclerotic lesions. Indeed, aggressive lipid lowering using obicetrapib, ezetimibe on atorvastatin background therapy resulted not only in inhibition of atherosclerosis progression but promoted true regression of total lesion size as well, despite mice being kept on a pro-atherogenic Western-type diet with a cholesterol content mimicking daily intake in humans. Mice that received this triple treatment had significantly more undiseased segments and fewer severe lesions. Similarly, as for atherosclerosis progression, the treatment effects on lesion area were predicted by the aggressive reduction in plasma non-HDL-C levels as illustrated by the strong association between the decreased non-HDL-C exposure and decreased lesion size during treatment (R = 0.783), indicating an important role of aggressive therapeutic cholesterol lowering in lesion regression. However, atherosclerosis regression was not only driven by non-HDL-C lowering, but by the increase in HDL-C concentrations as well, indicated by a significant correlation (R = 0.677). In eleven out of fifteen mice on triple treatment, lesion sizes were reduced to levels below those observed at baseline, indicating regression of atherosclerosis. In the remaining four mice, atherosclerosis progression was completely blocked versus baseline. Importantly, there was a strong increase in the percentage of undiseased segments in the triple treatment group, indicating resolution of atherosclerosis. More detailed analysis of atherosclerotic lesion composition revealed that double treatment and triple treatment contributed to more stable severe (type IV-V) lesions, with increased SMC and collagen content and decreased macrophage content.

In summary, we have demonstrated robust improvements of obicetrapib, ezetimibe, and their combination on plasma lipids in APOE∗3-Leiden.CETP mice. The efficacy of other potent and registered lipid-lowering interventions has been demonstrated before in this model, including the PCSK9 and ANGPTL3 monoclonal antibodies alirocumab and evinacumab, which both reduced non-HDL-C to a similar extent as obicetrapib and consequently improved atherosclerosis as well (20, 21, 24). Unlike PCSK9 and ANGPTL3 monoclonal antibodies, which require subcutaneous injection, obicetrapib and ezetimibe are administered orally, which may be a preferable strategy for patients. In a growing CVD patient population that requires alternative approaches for lipid lowering, these combination therapies are a promising strategy to reduce cardiovascular burden. The next-generation CETP inhibitor obicetrapib is currently under development for the treatment of dyslipidemia and CVD and has been shown to improve atherogenic lipoprotein levels (8, 9, 10, 37, 42). In this study, we elucidated the mechanism underlying the observed alterations in lipoprotein metabolism, demonstrating that obicetrapib and ezetimibe promote VLDL clearance from the circulation by upregulating LDL receptor expression without increasing hepatic lipid levels. Considering the benefits of obicetrapib and ezetimibe on atherosclerosis progression, as well as the strong beneficial effects of obicetrapib and ezetimibe when added to atorvastatin on plaque regression, this approach not only shows the potential to prevent but even reduce atherosclerotic lesions in the CVD patient population. Currently, obicetrapib is being studied in several phase 3 trials, including the PREVAIL trial (NCT05202509), which evaluates its potential to reduce the occurrence of major adverse cardiovascular events in patients with atherosclerotic CVD and for which results are expected in 2026 (https://clinicaltrials.gov/study/NCT05202509). Furthermore, the REMBRANDT phase 3 trial assesses the effects of obicetrapib and ezetimibe on coronary plaque characteristics using cardiovascular computed tomography angiography (CCTA) in patients at risk for CVD (NCT06305559) (43). These trials will reveal whether treatment with obicetrapib and ezetimibe translates into clinical benefit for the treatment of atherosclerotic CVD.

## Data availability

All data are contained within the manuscript.

## Supplemental data

This article contains supplemental data.

## Conflict of interest

The authors declare the following financial interests/personal relationships which may be considered as potential competing interests:

Co-authors Marc Ditmarsch, Mathijs de Kleer and John J. P. Kastelein are employees of NewAmsterdam Pharma, Naarden, the Netherlands. The funders had no role in data collection and raw data analysis.
