# Supplementary material for: Obicetrapib and ezetimibe enhance LDL receptor-mediated VLDL clearance and regress atherosclerosis on atorvastatin background
Source: J Lipid Res. 2026 Mar 25;67(5):101028. doi: 10.1016/j.jlr.2026.101028 (PMC13122311; doi:10.1016/j.jlr.2026.101028)
Supplement: Supplementary Materials [file mmc1.docx]

Supplementary material

**Obicetrapib alone and in combination with ezetimibe enhances LDL receptor-mediated VLDL-clearance and regresses atherosclerosis on atorvastatin background**

José A. Inia, Leo Zhang, Nanda Keijzer, Nicole Worms, Anita van Nieuwkoop-van Straalen, Marc Ditmarsch, Mathijs de Kleer, J. Wouter Jukema, John J.P. Kastelein, Michael Szarek, Anita M. van den Hoek, Geurt Stokman, Elsbet J. Pieterman, Hans M.G. Princen

# Supplementary material and methods

## Experimental design

Female mice were specifically selected for their dietary responsiveness and atherosclerosis development in time compared to males (1). Mice were group-housed in a temperature-controlled room with 12-hour light-dark cycle at 50−60% humidity and with free access to food and heat-sterilized water. Group sizes were determined a priori, using an effect size of 40-50% with a standard deviation of 35-50%, power of 80-90% and two-sided test with 95% confidence interval, resulting in group sizes of n = 10-15 mice per group. During the studies, mice received a Western-type diet (WTD) with 0.05% (w/w) cholesterol (S8854; Ssniff Spezialdiäten GmbH, Soest, Germany), unless otherwise specified. This cholesterol content is similar to the daily intake in humans (2). Mice received this diet without treatment (control) or this diet supplemented with obicetrapib, ezetimibe, atorvastatin, double treatment (obicetrapib and ezetimibe) or triple treatment (obicetrapib, ezetimibe and atorvastatin) as admixture to the diet. Mice were matched into groups based on age, body weight, total cholesterol, high-density lipoprotein cholesterol (HDL-C) and triglycerides. Intervention doses were selected based on doses used in humans, taking into account the 12.3 times faster metabolic rate in mice compared to humans (3). At several time points, after a 4-hour fasting period, blood was drawn from the tail vein into EDTA-coated tubes (Sarstedt, Nümbrecht, Germany). A schematic overview of the study set-up with all dietary and treatment conditions as well as measurements is provided in supplementary Fig. S1.

### Mechanism of action

To study the mechanism of action of obicetrapib and ezetimibe, mice (12-15 weeks old) received WTD + 0.05% cholesterol for a run-in period of four weeks, after which they were matched into groups (n = 10) to receive no intervention (control), obicetrapib (2 mg/kg/day), ezetimibe (1 mg/kg/day) or double treatment with obicetrapib and ezetimibe. After 5 weeks, very low-density lipoprotein (VLDL) production and *de novo* apolipoprotein B (ApoB) synthesis were determined and mice were sacrificed immediately afterwards. For experimental details see references (4–6).

Similarly, in a separate experiment in mice (11-15 weeks old), after a four-week run-in period and seven weeks of treatment, lipolytic activity of hepatic lipase (HL) and lipoprotein lipase (LPL) were determined. One week later, clearance of VLDL-like particles, plasma proprotein convertase subtilisin kexin type 9 (PCSK9) levels, hepatic low density lipoprotein receptor (LDLR) expression and liver lipids were determined, after which the mice were sacrificed. For experimental details see references (4–6).

### Atherosclerosis progression

After a four-week run-in period on WTD + 0.05% cholesterol, mice (8-13 weeks old) were matched into four groups that received no intervention (control), obicetrapib (2 mg/kg/day), ezetimibe (average 0.6 mg/kg/day) or the combination. The starting dose of ezetimibe (0.1 mg/kg/day) was chosen to yield a reduction in plasma cholesterol of approximately 20%, comparable to reductions observed in humans. After four weeks, this dosage did not sufficiently decrease plasma cholesterol and was increased to 0.2 mg/kg/day. The dosage was increased again to 1 mg/kg/day in week 10 and ultimately reduced to the final concentration of 0.6 mg/kg/day in week 16, a dosage that was maintained until the study endpoint and resulted in an average dose of 0.6 mg/kg/day. After 28 weeks of treatment, mice were sacrificed unfasted via gradual fill CO_2_ asphyxiation. Hearts were formalin-fixed and paraffin-embedded for histological analysis.

### Atherosclerosis regression

To study atherosclerosis regression, mice (8-14 weeks old) received the WTD with the addition of 0.3% cholesterol (Ssniff Spezialdiäten) for 12 weeks to induce atherosclerosis. After this run-in period, one group was sacrificed that served as baseline control. The other mice were matched into groups and switched to the WTD + 0.05% cholesterol. This diet was either given alone (control) or was supplemented with atorvastatin (5 mg/kg/day), obicetrapib and ezetimibe (2 mg/kg/day and 0.6 mg/kg/day, respectively) or the combination of atorvastatin, obicetrapib and ezetimibe. After 24 weeks of treatment, mice were sacrificed unfasted via gradual fill CO_2_ asphyxiation. Hearts were formalin-fixed and paraffin-embedded for histological analysis.

## Plasma and liver measurements

Endogenous CETP activity was determined as previously described (4). Cholesterol and triglyceride concentrations were determined using enzymatic colorimetric assays (Roche Diagnostics, Almere, the Netherlands). Plasma ApoA1 concentrations were determined as previously described (7) and plasma ApoB levels were determined using a mouse ApoB ELISA kit (Abcam, Cambridge, UK). HDL-C was determined after precipitation of ApoB-containing particles as described previously (8) and non-HDL-C was calculated by subtracting HDL-C from total plasma cholesterol. Non-HDL-C exposure was calculated as concentration × weeks. Lipoprotein profiles for cholesterol were measured in group-wise pooled plasma samples by fast protein liquid chromatography (FPLC) as described previously (9). Measurement of plasma PCSK9 levels was performed using the Quantikine mouse PCSK9 ELISA (MPC900; R&D Systems, Minneapolis, MN, USA) in accordance with the manufacturer’s protocol.

To determine the concentration of hepatic lipids, liver tissue of the lobus sinister lateralis hepatis was homogenized in phosphate-buffered saline (PBS) from n = 8 mice per group. Lipids were extracted and separated by high-performance thin-layer chromatography and analyzed with the ChemiDoc Touch Imaging System (Bio-Rad Laboratories Inc., Hercules, CA, USA) as described previously in detail (10). Hepatic triglycerides, cholesteryl ester and free cholesterol were quantified using Image-Lab software version 5.2.1. (Bio-Rad Laboratories) and expressed per mg of liver protein.

## VLDL production

For evaluation of VLDL production, mice were anesthetized by intraperitoneal injection with acepromazine-midazolam-fentanyl (6.25 mg/kg acepromazine (Produlab Pharma BV., Raamsdonksveer, the Netherlands), 6.25 mg/kg midazolam (Aurobindo, Baarn, the Netherlands) and 0.3125 mg/kg fentanyl (Hameln B.V., Leusden, the Netherlands)). A blood sample was taken directly after anesthesia and mice subsequently received an intravenous injection of 100 µL PBS containing Trans^35^S-labeled methionine/cysteine (3 µCi; ICN Biomedicals, Irvine, CA, USA) to measure *de novo* ApoB synthesis. Fifty-five minutes post-injection of the Trans^35^-label, mice were injected intravenously with 10% Triton WR1339 (500 mg/kg; Merck, Darmstadt, Germany) to completely block LPL activity and consequently VLDL clearance. Small blood samples (30 µL) were collected at t = 0 (prior to Triton WR1339 injection) and at t = 15, 30, 45 and 60 minutes post-injection and used to measure plasma triglycerides. At t = 60 minutes, mice were sacrificed by cervical dislocation and terminal blood was collected via cardiac puncture, from which VLDL was isolated by density-gradient ultracentrifugation. ^35^S-ApoB was measured in the VLDL fraction after ApoB-specific precipitation with isopropanol. *De novo* VLDL-ApoB production rate was calculated as the amount of 35S label incorporated in ApoB and expressed as disintegrations per minute (dpm) per mL of plasma per hour, as previously described in detail (4–6).

## Clearance of VLDL-like particles

For measurement of VLDL clearance, mice were treated with obicetrapib, ezetimibe or the combination for a total of eight weeks. In week 8, mice were fasted for four hours and injected into the tail vein with VLDL-mimicking particles (80 nm diameter) containing 1 µCi glycerol tri[^3^H]oleate ([^3^H]-TO) and 0.1 µCi [^14^C]-cholesteryl oleate ([^14^C]-CO) at a dose of 1 mg triglycerides per mouse. Blood samples (50 µL) were collected at 2, 5, 10 and 15 minutes post-injection. Fifteen minutes post-injection, mice were euthanized by cervical dislocation and perfused with 10 U/mL heparin in PBS for 5 minutes. [^3^H] and [^14^C] activities were counted in 5 µL plasma and corrected for total plasma volume. Liver, heart, spleen, quadriceps femoris muscle, perigonadal white adipose tissue (pWAT) and interscapular brown adipose tissue (BAT) were collected. Tissues were fully dissolved by overnight incubation with 500 µL Solvable (Perkin-Elmer, Wellesley, MA, USA) at 60°C, after which radioactivity was measured to determine uptake of [^3^H]-TO and [^14^C]-CO. Radioactive retention in tissues was measured as % of the injected dose. Plasma half-life of [^3^H]-TO and [^14^C]-CO-labeled VLDL-like particles was calculated from the slope after linear fitting of semi-logarithmic decay curves (4–6).

## Lipolytic activity

Seven weeks after the start of the intervention period, lipolytic activity of lipoprotein lipase (LPL) and hepatic lipase (HL) was determined. To this end, mice were fasted for four hours and injected with heparin (0.5 IU/g body weight; Leo Pharmaceutical Products BV, Weesp, the Netherlands) to liberate LPL from the endothelium and blood was collected 20 minutes post-injection. Post-heparin plasma triacylglycerol hydrolase activity was determined in the presence or absence of 1 M NaCl to determine LPL and HL activity. LPL activity was calculated as a portion of total lipase activity inhibited by 1 M NaCl.

## Hepatic LDL receptor expression

Approximately 50 mg of tissue of the lobus sinister lateralis hepatis was homogenized in lysis buffer containing 150 mM NaCl, 1 mM EDTA, 50 mM Tris-HCl (pH 7.4), 1% Igepal, 0.25% deoxycholate, 0.1% SDS, 1 mM PMSF, 1 mM Na_3_VO_4_ and complete protease inhibitor cocktail (Roche, Mannheim, Germany), using glass beads and a minibead beater (BioSpec Products, Bartlesville, OK, USA). Homogenates were centrifuged (13,000 rpm, 15 minutes at 4°C) and protein content of the supernatant was determined with the BCA Protein Assay Kit (ThermoFisher Scientific). Proteins (20 µg) in 2x SDS Laemmli Sample Buffer (Sigma-Aldrich, St. Louis, MO, USA) were separated on a 4-20% (w/v) SDS-PAGE gel (mini-Protean TGX stain-free precast gel; Bio-Rad Laboratories) and transferred onto Trans-blot Turbo mini PVDF blotting membranes (Bio-Rad Laboratories) on the Trans-blot Turbo Bio-Rad Machine (MIXED MW program). Membranes were blocked with 5% (w/v) milk powder in TBS with 0.1% Tween-20 for 1 hour at room temperature. Membranes were incubated overnight with goat anti-mouse LDLR antibody (#AF2255-1:1000; R&D Systems) or mouse anti-α-tubulin (T6199; 1:1000; Merck) in 5% milk in TBST. Subsequently, membranes were incubated with secondary antibodies (rabbit anti-goat-HRP; 5160-2504; 1:10000; Bio-rad Laboratories or horse anti-mouse-HRP; 7076S; 1:2000; Cell Signaling Technologies) in 5% milk in TBST and visualized with SuperSignal West Femto (ThermoFisher Scientific). Band intensity was determined using the ChemiDoc Touch Imaging system (Bio-Rad Laboratories) and normalized to α-tubulin (11,12).

## Histological assessment of atherosclerosis

Atherosclerotic lesion area and severity were assessed in the aortic root consistent with previously described protocols (8,13,14). In short, 5 µm cross-sections of the aortic root were made at 50 µm intervals and stained with hematoxylin-phloxine-saffron (HPS). Lesion area was assessed in four sections per mouse and classified in accordance with American Heart Association (AHA) criteria where 0 indicates the absence of lesions, i.e. undiseased segments, type I indicates early fatty streak, type II regular fatty streak, type III mild plaque, type IV moderate plaque and type V severe plaque (8,14).

## Histological assessment of lesion composition

For analysis of atherosclerotic lesion composition, all severe (type IV-V) lesions were further evaluated as described previously in detail (13,15). Briefly, a double immunostaining was performed using anti-α-smooth muscle actin (#61001; PROGEN Biotechnik GmbH) for smooth muscle cells (SMCs) and anti-LAMP2 (MA5-17861; Invitrogen) for macrophages. Subsequently, after incubation with secondary HRP-conjugated antibody (ab97057; Abcam), SMCs were visualized with Vina Green (Biocare Medical). Similarly, after incubation with secondary HRP-conjugated antibody (#P0260; Dako A/S, Glostrup, Denmark), macrophages were visualized with 3,3’-diaminobenzidine (DAB; Vector Laboratories). Slides were scanned with the Pannoramic 500 scanner (3DHISTECH Ltd., Budapest, Hungary) and analyzed using customized macros in ImageJ (version 1.53; NIH). Coverslips were detached overnight in xylene and a Sirius Red staining was performed next to visualize collagen. Customized macros in ImageJ were applied to quantify collagen content and necrotic core content, which is defined as a pool of accumulated cellular debris and extracellular lipids, including cholesterol clefts. A plaque stability index was calculated by dividing plaque stabilizing factors (the sum of SMC content and collagen content) by plaque destabilizing factors (the sum of macrophage content and necrotic core content). This ratio is derived from human atherosclerosis pathology, where vulnerable lesions are characterized by increased macrophage content, large necrotic cores and thin fibrous caps (16).

## Statistical analysis

Correlations between total (non-)HDL-C exposure and atherosclerotic lesion area were determined by Spearman’s rank-order correlation test after normalization of atherosclerotic lesion area by cubic root transformation (lesion area^(1/3)^).

An analysis of covariance (ANCOVA) was performed to test for group differences in atherosclerotic lesion area with total HDL-C exposure and non-HDL-C exposure as covariates. To determine the presence of collinearity between the explanatory variables, variance inflation factor (VIF) and condition index (CI) were calculated. Values of VIF>5 and CI>10 were used as cutoffs for collinearity (17,18). Data are presented as mean±SEM. Two-sided p-values are reported and a p-value<0.05 was considered statistically significant.

The Bliss model of independence (19) was applied to determine whether treatment with obicetrapib and ezetimibe has a synergistic effect on non-HDL-C reduction. To facilitate statistical testing, the logarithm of the “surviving fraction” of non-HDL-C during treatment, i.e., 1 – the percent reduction, was analyzed in an ANCOVA model accounting for changes in the control group (20) with treatment group as fixed effect and baseline non-HDL-C as covariate. With these conventions, the hypotheses are:

H_0_: log(μ_obicetrapib_) + log(μ_ezetimibe_) - log(μ_obicetrapib+ezetimibe_) - log(μ_control_) = 0

H_1_: log(μ_obicetrapib_) + log(μ_ezetimibe_) - log(μ_obicetrapib+ezetimibe_) - log(μ_control_) > 0

where µ is the mean surviving fraction for the indicated treatment group. In the current application, the null hypothesis specifies that the obicetrapib and ezetimibe combination reduce non-HDL-C independently, whereas the alternative hypothesis specifies a synergistic effect. Through reverse transformation, the estimated synergy and associated two-sided 95% confidence interval was calculated, along with a one-sided p-value.

Possible synergistic effects of the combination of obicetrapib and ezetimibe on atherosclerotic lesion area was analyzed in an ANCOVA model with treatment with obicetrapib, ezetimibe and their combination as fixed effects and baseline non-HDL-C as covariate. Evidence of synergy was tested by the two-sided interaction effect p-value.

Linear regression analysis was performed to determine the contribution of the cumulative decrease in non-HDL-C to regression of atherosclerosis.

# Supplemental figures and tables

**Supplemental figure 1. Study set-up.** Mice were placed on a run-in diet to induce hyperlipidemia and were matched into equal groups at t = week 0. Upon randomization, mice continued to be fed the Western-type diet with 0.05% cholesterol (control), or this diet supplemented with obicetrapib, ezetimibe, the combination of obicetrapib and ezetimibe or obicetrapib and ezetimibe on top of atorvastatin (dosages indicated as mg/kg body weight/day). Asterisks indicate timepoints at which body weight, food intake and plasma parameters were determined. Abbreviations: ApoB: apolipoprotein B; HL: hepatic lipase; LDLR: low density lipoprotein receptor; LPL: lipoprotein lipase; PCSK9: proprotein convertase subtilisin kexin type 9; VLDL: very low density lipoprotein; WTD: Western-type diet.


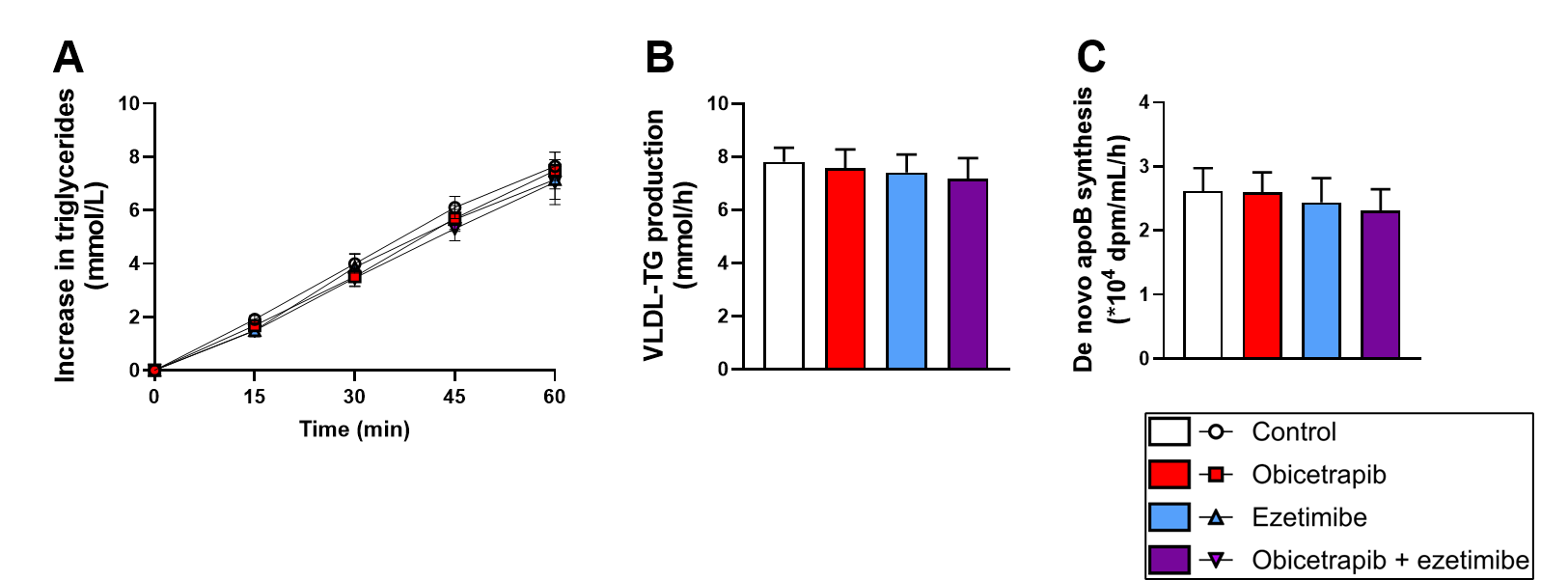


**Supplemental figure 2. VLDL production and *de novo* ApoB synthesis are not affected by obicetrapib and ezetimibe.** After five weeks of treatment with obicetrapib, ezetimibe or the combination, 4 hour fasted mice were injected with Tran^35^S-label and Triton, after which blood was collected in the following 60 minutes. Plasma VLDL-triglycerides (A) were plotted and used to calculate the rate of triglyceride production (B) from the slope of the individual curves. Plasma was collected 90 minutes after Triton injection and VLDL was isolated by ultracentrifugation to assess the rate of newly synthesized ApoB (C). Data are presented as mean ± SEM (n = 10 per group).


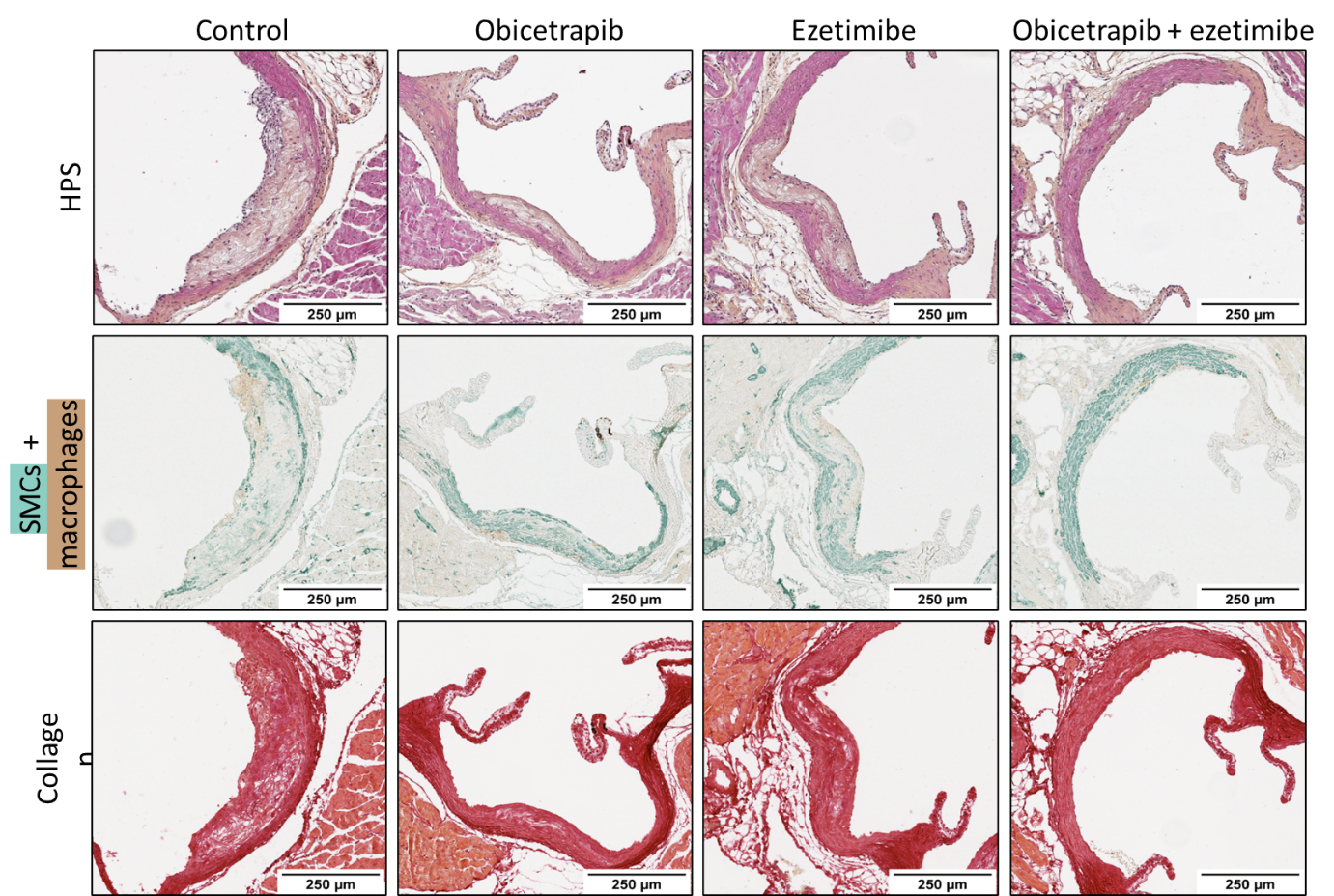


**Supplemental figure 3. Composition of atherosclerotic plaques in the atherosclerosis progression study.** Composition of severe (type IV-V) atherosclerotic plaques was analyzed in aortic root cross-sections stained with hematoxylin-phloxine-saffron (HPS), double-stained with α-actin for smooth muscle cells (SMCs; Vina Green) and LAMP2 (M3/84) for macrophages (DAB, brown), or Sirius Red for collagen and necrosis content.


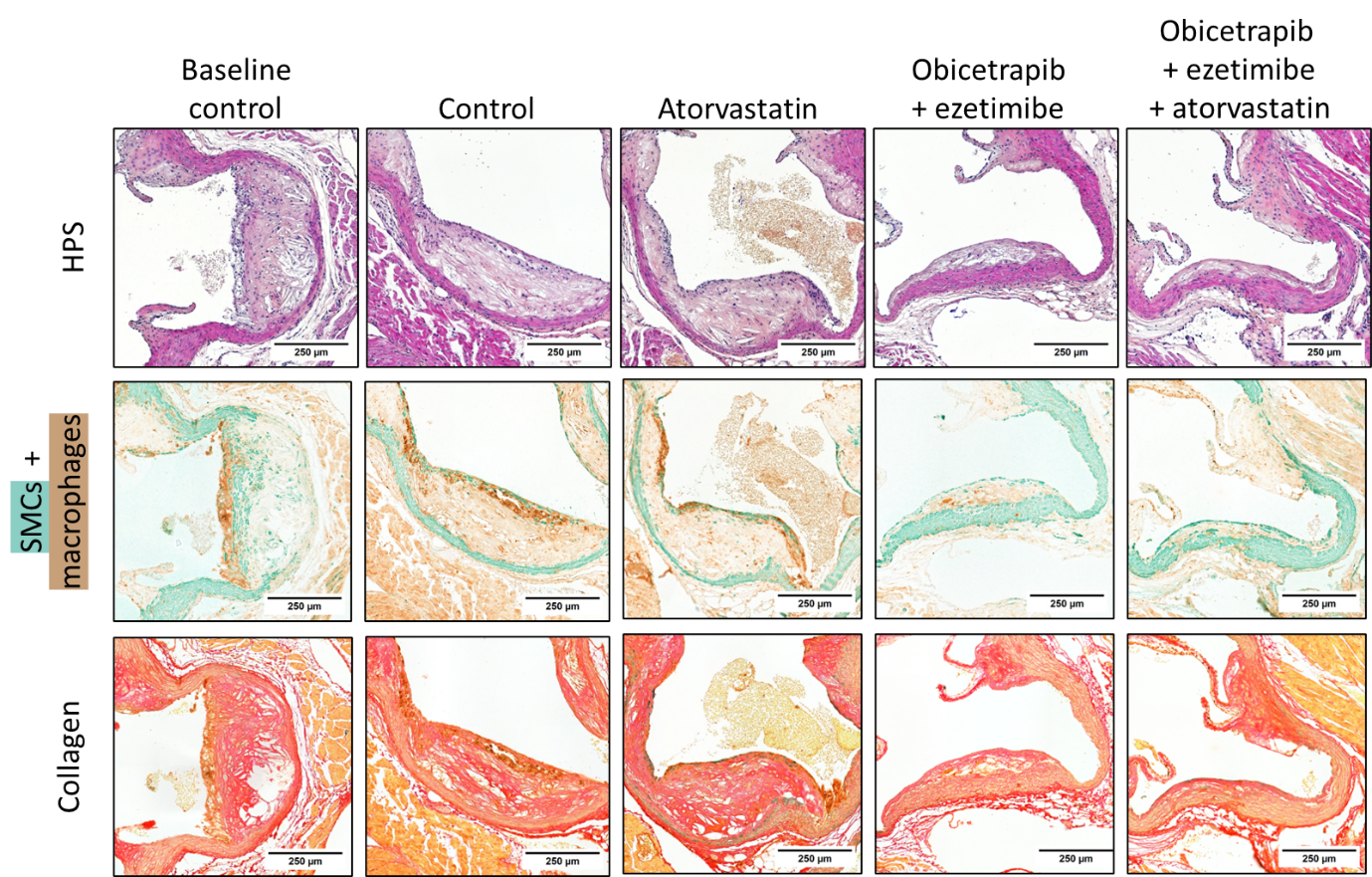


**Supplemental figure 4. Composition of atherosclerotic plaques in the atherosclerosis regression study.** Composition of severe (type IV-V) atherosclerotic plaques was analyzed in aortic root cross-sections stained with hematoxylin-phloxine-saffron (HPS), double-stained with α-actin for smooth muscle cells (SMCs; Vina Green) and LAMP2 (M3/84) for macrophages (DAB, brown), or Sirius Red for collagen and necrosis content.

# References

1. Van Vlijmen BJM, Van ’t Hof HB, Mol MJ, Van der Boom H, Van der Zee A, Frants RR, et al. Modulation of very low density lipoprotein production and clearance contributes to age- and gender- dependent hyperlipoproteinemia in apolipoprotein E3-Leiden transgenic mice. Journal of Clinical Investigation. 1996;97(5):1184–92.

2. Xu Z, McClure ST, Appel LJ. Dietary Cholesterol Intake and Sources among U.S Adults: Results from National Health and Nutrition Examination Surveys (NHANES), 2001−2014. Nutrients. 2018;10(6).

3. Nair AB, Jacob S. A simple practice guide for dose conversion between animals and human. Journal of Basic and Clinical Pharmacy. 2016;7(2):27–31.

4. Kühnast S, Louwe MC, Heemskerk MM, Pieterman EJ, Van Klinken JB, Van den Berg SAA, et al. Niacin Reduces Atherosclerosis Development in APOE*3Leiden.CETP Mice Mainly by Reducing NonHDL-Cholesterol. PLoS ONE. 2013 Jun 19;8(6):66467. PubMed PMID: 23840481.

5. Pouwer MG, Pieterman EJ, Chang SC, Olsen GW, Caspers MPM, Verschuren L, et al. Dose Effects of Ammonium Perfluorooctanoate on Lipoprotein Metabolism in APOE*3-Leiden.CETP Mice. Toxicological Sciences: an official journal of the Society of Toxicology. 2019;168(2):519–34.

6. Stokman G, Van den Hoek AM, Denker Thorbekk D, Pieterman EJ, Skovgård Veidal S, Basta B, et al. Dual targeting of hepatic fibrosis and atherogenesis by icosabutate, an engineered eicosapentaenoic acid derivative. Liver International. 2020;40(11):2860–76. PubMed PMID: 32841505.

7. De Haan W, Van der Hoogt CC, Westerterp M, Hoekstra M, Dallinga-Thie GM, Princen HMG, et al. Atorvastatin increases HDL cholesterol by reducing CETP expression in cholesterol-fed APOE*3-Leiden.CETP mice. Atherosclerosis. 2008;197(1):57–63.

8. Kühnast S, Van der Tuin SJL, Van der Hoorn JWA, Van Klinken JB, Simic B, Pieterman EJ, et al. Anacetrapib reduces progression of atherosclerosis, mainly by reducing non-HDL-cholesterol, improves lesion stability and adds to the beneficial effects of atorvastatin. European Heart Journal. 2015;36(1):39–48.

9. Westerterp M, Van der Hoogt CC, De Haan W, Offerman EH, Dallinga-Thie GM, Jukema JW, et al. Cholesteryl ester transfer protein decreases high-density lipoprotein and severely aggravates atherosclerosis in APOE*3-Leiden mice. Arteriosclerosis, Thrombosis, and Vascular Biology. 2006;26(11):2552–9.

10. Post SM, De Crom R, Van Haperen R, Van Tol A, Princen HMG. Increased fecal bile acid excretion in transgenic mice with elevated expression of human phospholipid transfer protein. Arteriosclerosis, Thrombosis, and Vascular Biology. 2003;23(5):892–7.

11. Kühnast S, Van Der Hoorn JWA, Pieterman EJ, Van den Hoek AM, Sasiela WJ, Gusarova V, et al. Alirocumab inhibits atherosclerosis, improves the plaque morphology, and enhances the effects of a statin. Journal of Lipid Research. 2014;55(10):2103–12.

12. Suchowerska AK, Stokman G, Palmer JT, Coghlan PA, Pieterman EJ, Keijzer N, et al. A Novel, Orally Bioavailable, Small-Molecule Inhibitor of PCSK9 With Significant Cholesterol-Lowering Properties In Vivo. Journal of Lipid Research. 2022;63(11).

13. Pouwer MG, Pieterman EJ, Worms N, Keijzer N, Jukema JW, Gromada J, et al. Alirocumab, evinacumab, and atorvastatin triple therapy regresses plaque lesions and improves lesion composition in mice. Journal of Lipid Research. 2020;61(3):365.

14. Kühnast S, Van Der Hoorn JWA, Van Den Hoek AM, Havekes LM, Liau G, Jukema JW, et al. Aliskiren inhibits atherosclerosis development and improves plaque stability in APOE*3Leiden.CETP transgenic mice with or without treatment with atorvastatin. Journal of Hypertension. 2012;30(1):107–16.

15. Pouwer MG, Pieterman EJ, Verschuren L, Caspers MPM, Kluft C, Garcia RA, et al. The BCR-ABL1 Inhibitors Imatinib and Ponatinib Decrease Plasma Cholesterol and Atherosclerosis, and Nilotinib and Ponatinib Activate Coagulation in a Translational Mouse Model. Frontiers in Cardiovascular Medicine. 2018;5(55).

16. Libby P, Sasiela W. Plaque stabilization: Can we turn theory into evidence? The American Journal of Cardiology. 2006;98(11A).

17. Belsley DA. A guide to using the collinearity diagnostics. Comp Sci Econ Manage. 1991;4:33–50.

18. Menard S. Applied logistic regression analysis (Sage university paper series on quantitative application in the social sciences, series no. 106). Thousand Oaks, CA: Sage; 1995.

19. Bliss CI. The toxicity of poisons applied jointly. Annals of Applied Biology. 1939;26(3):585–615.

20. Demidenko E, Miller TW. Statistical determination of synergy based on Bliss definition of drugs independence. PLOS ONE. 2019;14(11):e0224137.
